# Cross-scale modeling reveals a TFRC-driven immunosuppressive macrophage niche in cervical cancer

**DOI:** 10.3389/fimmu.2026.1872944

**Published:** 2026-07-17

**Authors:** Yusha Chen, Ling Wang, Suyu Li, Jimiao Huang, Leilei Zhu, Xiqi Huang, Xiangqin Zheng, Diling Pan, Chuanzhong Huang

**Affiliations:** 1 Cervical disease diagnosis and treatment health center, Fujian Maternity and Child Health Hospital College of Clinical Medical for Obstetrics & Gynecology and Pediatrics, Fujian Medical University, Fuzhou, China; 2 Laboratory of Immuno-Oncology, Clinical Oncology School of Fujian Medical University, Fujian Cancer Hospital, Fuzhou, China; 3 Fujian Key Laboratory of Translational Cancer Medicine, Fuzhou, China; 4 Department of Radiation Oncology, Fujian Maternity and Child Health Hospital, College of Clinical Medical for Obstetrics & Gynecology and Pediatrics, Fujian Medical University, Fuzhou, China; 5 Department of Gynecology, Fujian Maternity and Child Health Hospital College of Clinical Medical for Obstetrics & Gynecology and Pediatrics, Fujian Medical University, Fuzhou, China; 6 Department of Pathology, Fujian Maternity and Child Health Hospital College of Clinical Medical for Obstetrics & Gynecology and Pediatrics, Fujian Medical University, Fuzhou, China

**Keywords:** cervical cancer, macrophage polarization, prognostic signature, TFRC, tumor-associated macrophages

## Abstract

**Background:**

The functional plasticity of tumor-associated macrophages (TAMs) is a critical determinant of the immunosuppressive microenvironment in cervical cancer, yet its integration into actionable prognostic frameworks remains limited. This study aimed to establish a TAM polarization-centered model and elucidate the mechanisms of underlying tumor-immune crosstalk.

**Methods:**

Bulk transcriptomics from The Cancer Genome Atlas (TCGA) were integrated with single-cell RNA sequencing (scRNA-seq) data (GSE208653). By combining weighted gene co-expression network analysis (WGCNA) with a multi-algorithm machine learning framework, a prognostic signature was constructed and independently validated in the Gene Expression Omnibus (GEO) GSE52903 cohort. Single-cell analysis resolved the cellular origins of signature genes, prioritizing tumor-enriched genes for validation. Protein-level expression was verified via immunohistochemistry (IHC) in a paired clinical cohort (n=39). Functional validation of the core gene was performed *in vitro* using cervical cancer cell lines co-cultured with THP-1-derived macrophages. Polarization was assessed via reverse transcription-quantitative polymerase chain reaction (RT-qPCR), Western blot (WB), enzyme-linked immunosorbent assay (ELISA), flow cytometry, and multiplex immunofluorescence (mIF).

**Results:**

A robust five-gene prognostic signature (TP73, TFRC, SHC1, SCD, and PFKFB3) was developed, effectively stratifying patient survival. High risk scores correlated with a suppressed antitumor immune landscape and diminished predicted chemosensitivity to agents such as cisplatin. Single-cell analysis and IHC confirmed transferrin receptor (TFRC) as a tumor-intrinsic factor that is progressively upregulated during cervical carcinogenesis and enhances pro-M2 signaling. *In vitro* co-culture assays demonstrated that tumor-derived TFRC actively orchestrates an immunosuppressive M2-like macrophage niche, driving phenotypic shifts and pro-tumorigenic cytokine secretion, characterized by elevated interleukin-10 (IL-10) and reduced TNF-α. RT-qPCR analysis of 40 clinical specimens further confirmed a significant positive correlation between TFRC and the M2 marker Arg-1 at the mRNA level (r = 0.4961, P = 0.0011).

**Conclusions:**

This study establishes a cross-scale, biologically interpretable prognostic model linking macrophage plasticity to clinical outcomes. We identify TFRC as a pivotal metabolic-immune node through which tumor-intrinsic iron metabolism orchestrates an immunosuppressive niche, providing a foundation for novel therapeutic strategies in cervical cancer.

## Introduction

Cervical cancer remains a global health priority, representing a leading cause of malignancy-related morbidity and mortality among women worldwide (1). Despite the implementation of comprehensive screening and multimodal therapeutic strategies, the clinical outlook for patients with advanced or recurrent disease remains suboptimal (2, 3). The substantial clinical heterogeneity observed among patients with comparable clinicopathological features necessitates the discovery of precise molecular biomarkers to refine prognostic stratification and facilitate individualized clinical management (4).

The tumor microenvironment (TME) is a primary determinant of cervical cancer progression and therapeutic response (5–7). Within this complex ecosystem, tumor-associated macrophages (TAMs) constitute a dominant and highly plastic immune cell population (8, 9). TAMs exhibit dynamic polarization along a functional spectrum, ranging from pro-inflammatory, anti-tumor M1-like phenotypes to immunosuppressive, pro-tumor M2-like states (10–13). A skewed M2-like polarization—or a diminished M1/M2 ratio—consistently correlates with inferior survival and therapeutic resistance in cervical cancer (14). Emerging evidence underscores that these immunosuppressive TAMs are pivotal drivers of chemoresistance and active immune evasion (15–17), yet the specific transcriptional programs governing this TAM-mediated therapeutic failure remain to be fully integrated into robust clinical models.

While recent computational advances have enabled the characterization of immune-related signatures in cervical cancer, most existing models describe immune infiltration at a general level, failing to explicitly capture the functional heterogeneity and metabolic-immune crosstalk inherent to macrophage polarization (18–23). Furthermore, many reported signatures lack the rigorous cross-scale validation—bridging bulk-level trends with single-cell resolution and experimental evidence—required for translational applicability (18–20). Consequently, there is a compelling need for a prognostic framework that systematically incorporates macrophage plasticity and is validated through an integrated computational and functional pipeline.

To address these gaps, we developed an integrative cross-scale framework. By combining large-scale transcriptomic deconvolution with weighted gene co-expression network analysis (WGCNA), we identified key macrophage-associated gene modules. A multi-algorithm machine learning pipeline—comprising random forest, SVM-based recursive feature elimination (SVM-RFE), and LASSO Cox regression—was employed to construct a robust five-gene prognostic signature. To resolve the cellular architecture of this signature, we utilized single-cell transcriptomic analysis to pinpoint the precise cellular origins of candidate genes and infer the intercellular communication networks driving the immunosuppressive niche. Finally, we selected TFRC, a nodal metabolic-immune component identified by our model, for functional validation in tumor-macrophage co-culture systems to confirm its role in orchestrating M2-like polarization and facilitating immune evasion.

Together, this study establishes a biologically interpretable prognostic framework grounded in macrophage plasticity and provides novel insights into how tumor-intrinsic iron metabolism reshapes the immunosuppressive niche in cervical cancer.

## Material and methods

### Data acquisition and processing

Clinical data and RNA sequencing profiles for cervical squamous cell carcinoma and endocervical adenocarcinoma (CESC) were obtained from The Cancer Genome Atlas (TCGA; https://portal.gdc.cancer.gov). The study included 291 CESC samples with complete prognostic annotations, alongside 10 normal cervical tissue samples from the GTEx database serving as non-malignant controls. To mitigate batch effects and technical variability, raw sequencing data were normalized, and fragments per kilobase of transcript per million mapped reads (FPKM) values were converted to transcripts per kilobase million (TPM). For external validation, microarray data (GSE52903) were retrieved from the Gene Expression Omnibus (GEO), comprising expression profiles and survival data from 55 CESC patients and 17 healthy exocervical controls. To resolve the specific cellular origins of the identified prognostic genes at single-cell resolution, the scRNA-seq dataset GSE208653 was utilized. Eight representative samples spanning the cervical carcinogenesis continuum were selected: two human papillomavirus (HPV)-negative normal, two HPV-positive normal, two high-grade squamous intraepithelial lesion (HSIL), and two invasive squamous cell carcinoma (SCC) samples. All bioinformatic analyses were conducted using R software (version 4.3.3; https://www.r-project.org).

### Macrophage infiltration and survival analysis

The relative proportions of M1 and M2 macrophages within the TCGA-CESC cohort were quantified using the CIBERSORT (24) algorithm with its default leukocyte signature matrix. To evaluate prognostic relevance, patients were stratified into high- and low-infiltration groups based on their individual M1, M2, and M1/M2 macrophage ratios. The optimal cutoff thresholds for this stratification were objectively determined using the survcutpoint function within the survminer R package (v0.5.0). To maintain analytical robustness, samples with undetectable levels of both M1 and M2 macrophages were excluded from downstream analyses. Kaplan-Meier survival curves were subsequently generated using the survival (v3.7.0) package to compare clinical outcomes between the stratified groups, thereby assessing the prognostic significance of macrophage polarization states in cervical cancer.

### Weighted gene co-expression network analysis

To identify transcriptional programs associated with macrophage polarization, we applied WGCNA (v1.73) (25) to the TCGA-CESC dataset. Prior to WGCNA, hierarchical clustering of all samples was performed using Euclidean distance and average linkage to screen for outliers. No extreme outlier samples were identified, and all samples were retained for downstream analysis. The dichotomized M1/M2 ratio (high vs. low) served as the primary clinical trait. Pearson correlation coefficients across all gene pairs were calculated to generate a similarity matrix, which was subsequently transformed into an adjacency matrix. An optimal soft-thresholding power (β= 6) was applied to achieve a scale-free network topology (R^2^ > 0.85). Gene modules were clustered using a merge cut height of 0.4, and module-trait relationships were evaluated to isolate co-expression networks significantly correlated with the M1/M2 polarization status. For all genes within the prioritized module, Module Membership (MM), defined as the Pearson correlation between each gene’s expression and the module eigengene, and Gene Significance (GS), defined as the Pearson correlation between each gene’s expression and the M1/M2 ratio trait, were calculated. Only genes satisfying both |MM| > 0.8 and |GS| > 0.2 were retained as high-confidence module members for downstream analysis.

### Cox regression analysis, consensus clustering, and subtype characterization

Univariate Cox regression was applied to genes within the prioritized WGCNA module to identify prognostic candidates, yielding 121 survival-associated genes (*P* < 0.05). Based on the expression profiles of these genes, consensus clustering (via the ConsensusClusterPlus (v1.66.0) package (26)) optimally stratified patients into two distinct molecular subtypes (K = 2). Clinical outcomes between these subtypes were compared using Kaplan-Meier survival analysis. To characterize the tumor microenvironment (TME), stromal and immune infiltration scores were quantified utilizing the ESTIMATE (27) algorithm. Differentially expressed genes (DEGs) between the subtypes (FDR < 0.05, determined via the limma (v3.58.1) package) were subsequently subjected to Gene Ontology (GO) and Kyoto Encyclopedia of Genes and Genomes (KEGG) functional enrichment analyses. Furthermore, the expression profiles of human leukocyte antigen (HLA)-related genes were compared across subtypes to evaluate differences in antigen-presentation capacity.

### Protein-protein interaction network and hub gene identification

A PPI network was constructed from DEGs using the STRING database (confidence score ≥ 0.4) and visualized in Cytoscape. Hub genes were identified via the cytoHubba plugin, with the top 20 nodes ranked by Maximal Clique Centrality (MCC). These hub genes were considered key regulatory factors and further evaluated for prognostic significance.

### Machine learning for hub gene selection

To isolate key prognostic hub genes, we implemented a feature selection pipeline comprising three distinct machine learning algorithms: Gaussian Mixture Model (GMM), Support Vector Machine-Recursive Feature Elimination (SVM-RFE), and Random Forest. Specifically, GMM (via the SimDesign (v2.18) package (27)) was utilized to model gene expression distributions; SVM-RFE (28) (utilizing the e1071 (v1.7.16), kernlab (v0.9.33), and caret (v7.0.1) packages) was applied to iteratively eliminate low-impact features; and Random Forest (via the randomForest (v4.7.1.2) package (29)) was employed to optimize classification accuracy. Genes independently identified by all three algorithms were intersected to define a robust panel of differentially expressed core-set genes (DECSGs). The algorithmic overlap was subsequently visualized using the VennDiagram package (30).

### Construction and validation of the prognostic signature

A prognostic risk model was developed using LASSO Cox regression based on DECSGs expression in the TCGA-CESC cohort. The model was trained with cross-validation via the “cv.glmnet” function (glmnet v4.1.8) to determine the optimal lambda. Gene coefficients were extracted using the “coef” function and incorporated into the risk score formula: Risk Score = Σ(Coefficient_i_ × Expression_i_). Patients were stratified into high- and low-risk groups based on the optimal cut-point determined using the surv_cutpoint function (survminer package v0.4.9). Kaplan-Meier analysis compared overall survival (OS) between groups. Time-dependent receiver operating characteristic (ROC) analysis was performed using the “timeROC” (v0.4) package (31). The model was validated in an independent cohort (GSE52903) to confirm generalizability. The proportional hazards assumption for the five-gene Cox model was formally assessed using the Schoenfeld residuals test (cox.zph() function, survival package v3.7.0).

### Prognostic nomogram construction

A nomogram integrating patient age and the five-gene risk score was developed to predict 1-, 3-, and 5-year OS in the TCGA-CESC dataset. Multivariate Cox regression confirmed both variables as independent prognostic factors. Predictive performance was assessed using ROC curves and calibration plots. Decision curve analysis (DCA) evaluated clinical utility across threshold probabilities.

### Immune infiltration and checkpoint expression analysis

The tumor immune microenvironment was comprehensively characterized using single-sample gene set enrichment analysis (ssGSEA), implemented via the gsva() function in the GSVA R package (v1.48.0) with the method = “ssgsea” option and immune cell-specific gene signatures curated by Bindea et al. (2013) (32). This analysis quantified the relative infiltration levels of 25 distinct immune cell subsets encompassing both adaptive and innate lineages in the TCGA-CESC cohort. Differences in ssGSEA scores between the low- and high-risk groups were compared using the Wilcoxon rank-sum test. Furthermore, Pearson correlation analysis was applied to evaluate the associations between the model-derived risk score and the expression levels of key immune checkpoint molecules (e.g., PD-1, CTLA-4, LAG-3).

### Drug sensitivity prediction

The relationship between the risk score and chemotherapeutic response was evaluated using the “oncoPredict” R package (v0.2) (33). The analysis was based on the bulk tissue pharmacogenomic data from the Genomics of Drug Sensitivity in Cancer (GDSC) database (v2; https://www.cancerrxgene.org/) (34). The estimated half-maximal inhibitory concentration (IC_50_) values for each compound were compared between the high- and low-risk groups using the Wilcoxon test. Compounds with significantly lower IC_50_ values in the high-risk group (*P* < 0.05) were identified as candidate agents with potential therapeutic efficacy for this patient subgroup.

### Single-cell RNA sequencing data processing and analysis

For the GSE208653 dataset, data preprocessing, quality control (excluding cells with >20% mitochondrial or >3% hemoglobin transcripts), normalization, and Uniform Manifold Approximation and Projection (UMAP) dimensionality reduction were performed utilizing Seurat (v5.2.1). Ten major cell lineages were annotated based on the expression of canonical marker genes. To bridge the bulk-derived findings with single-cell resolution, we evaluated the expression patterns of the five genes constituting the prognostic signature (TP73, TFRC, SHC1, SCD, and PFKFB3). Expression density analysis via Nebulosa (v1.12.1) (35) revealed that TFRC was uniquely and predominantly enriched within the malignant epithelial compartment; consequently, TFRC was prioritized for subsequent downstream functional interrogation. Subsequently, the myeloid compartment was subclustered, and cells were computationally classified into M1 or M2 macrophage phenotypes utilizing AUCell (v1.24.0) (36). To investigate the transcriptomic features and intercellular crosstalk associated with TFRC, malignant epithelial cells were stratified into TFRC-high and TFRC-low subgroups, corresponding to the top and bottom 30% of expression levels, respectively. Differentially expressed genes (DEGs) between these strata were identified using the Wilcoxon rank-sum test (adjusted P < 0.05, |log2FC| > 0.25), and functional enrichment of the upregulated DEGs was evaluated through Gene Ontology (GO) analysis via clusterProfiler (v4.10.1). Finally, CellChat (v1.6.1) (37) was employed to infer and compare the secreted signaling networks originating from the TFRC-high and TFRC-low malignant clones toward the M2 macrophage populations.

### *In vitro* assays

Human cervical cancer cell lines (SiHa, C-33A, AV3, and Caski) were obtained from the Cell Bank of the Chinese Academy of Sciences (Shanghai, China). Cells were cultured in RPMI-1640 or DMEM (Hyclone) supplemented with 10% fetal bovine serum (Gibco) and incubated at 37°C in a humidified atmosphere with 5% CO2 incubator. All cell lines were routinely tested for mycoplasma contamination (Lonza). For functional assays, full-length TFRC cDNA cloned into the pcDNA3.1 vector or TFRC-targeting siRNAs (synthesised and sequence-verified by Obio) were transfected using Lipofectamine 3000 (Invitrogen, #L3000015) according to the manufacturer’s instructions. Transfection efficiency was assessed 48 hours post-transfection.

### Transwell-based macrophage co-culture assay

THP-1 monocytes were seeded at 5×105 cells/mL and differentiated into macrophages by treatment with 100 ng/mL phorbol 12-myristate 13-acetate (PMA, MCE, #HY-18739) for 24 hours, followed by a 24-hour resting period in PMA-free complete medium. A Transwell co-culture system was established using 6.5 mm inserts with 0.4 µm pores. Transfected cervical cancer cells were seeded in the upper chambers, and the inserts were placed into 24-well plates containing the fully adherent, differentiated THP-1 macrophages. After 24–48 hours of co-culture, macrophages and supernatants were harvested separately for subsequent analyses.

### Protein, RNA, and cytokine analyses

Whole-cell lysates were prepared using cell lysis buffer, and protein concentrations were determined by the BCA method. Equal amounts of proteins were separated by SDS-PAGE and transferred to PVDF membranes (Millipore). The following primary antibodies were used for immunoblotting: TFRC (Abcam, #ab214039, 1:1000), CD68 (ABclonal, #A23205), CD86 (ABclonal, #A16805), CD206 (ABclonal, #A26948), and β-actin (CST, #8457).

Total RNA was extracted using the standard Trizol method. Following reverse transcription, mRNA abundance was evaluated via RT-qPCR using the 2^-ΔΔCt^ method, with β-actin serving as the internal control (primer sequences are listed in **Supplementary Table 1**). Cytokine concentrations in the co-culture supernatants were measured using commercial ELISA kits (ZCi BIO, China) following the manufacturer’s protocols, with optical density read at 450 nm.

### Flow cytometry and multiplex immunofluorescence

After co-culture, THP-1-derived macrophages were gently detached with 5 mM EDTA/PBS. Up to 1×10^6^ cells per sample were incubated with Zombie-NIR fixable viability dye (Biolegend, #423105), blocked with 10% human Fc-block, and stained with the following fluorochrome-conjugated antibodies: CD68-APC (Biolegend, #333809), CD86-FITC (Biolegend, #374203), and CD206-PE (Biolegend, #321105). Data were acquired on a BD FACSCanto II flow cytometer and analyzed using FlowJo v10. M1 and M2 subsets were defined as CD68^+^CD86^+^ and CD68^+^CD206^+^, respectively.

Multiplex immunofluorescence (mIF) was performed on macrophages using a dual-plus-triple fluorescence kit (Aifang Biotech, #AFIHC023) with sequential staining for CD68, CD86, and CD206. Nuclei were counterstained with DAPI, and images were acquired via inverted fluorescence microscopy.

### Clinical sample collection and immunohistochemistry

The study included 39 cervical cancer patients, from whom formalin-fixed, paraffin-embedded (FFPE) tumor tissues and corresponding adjacent non-malignant tissues were obtained at Fujian Maternity and Child Health Hospital between April 2018 and August 2025. The study protocol was approved by the Institutional Ethics Committee (Approval No. 2026KY131-02) and conducted in accordance with the Declaration of Helsinki. Authorization for the research use of resected tumor tissues was obtained from all patients.

IHC staining for TFRC (Abcam, #ab214039, 1:100) was performed on FFPE sections following standard protocols. TFRC protein expression was semi-quantitatively interpreted by two independent pathologists blinded to the clinical data. Staining intensity was scored as 0 (negative), 1 (weak), 2 (moderate), or 3 (strong). Discrepant scores were resolved through consensus review. Additionally, fresh tumor tissue specimens from an independent cohort of 40 cervical cancer patients were collected for RT-qPCR quantification of TFRC and Arg-1 mRNA expression levels.

### Statistical analysis

All experiments were independently repeated at least three times. Statistical analyses were performed using R version 4.3.3 and GraphPad Prism 9.0. Data are expressed as mean standard deviation (SD). For the comparison of TFRC IHC scores between paired tumor and adjacent tissues (n=39), the Wilcoxon signed-rank test was employed. Pearson correlation analysis was applied to assess the co-expression relationship between TFRC and Arg-1 in the clinical cohort. For other two-group comparisons, a Student’s t-test was used. Statistical significance was defined as a two-sided *P* < 0.05.

## Results

### 1 Identification of a macrophage polarization-associated gene module via WGCNA

**Figure 1:**
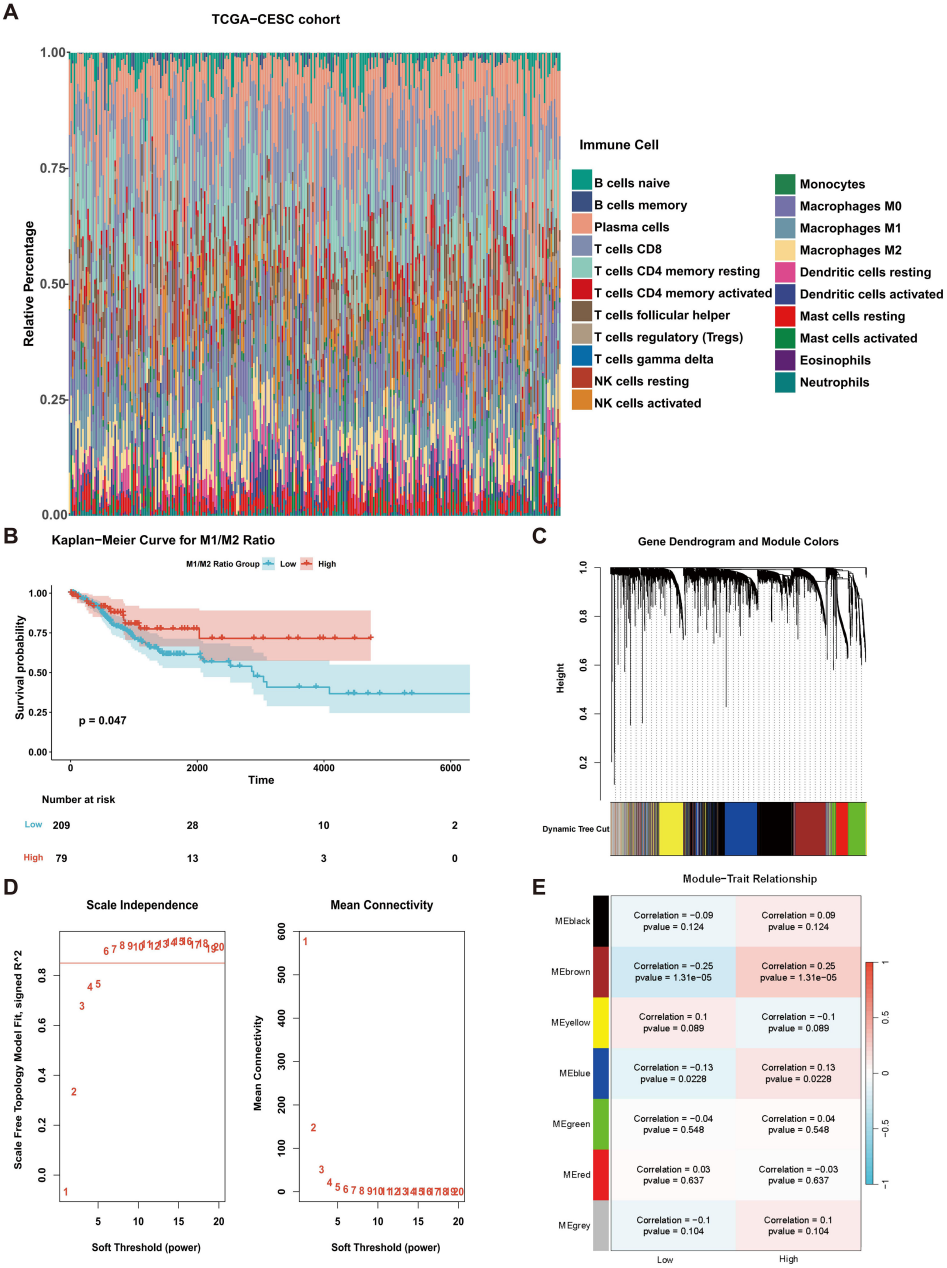
Prognostic Impact of Macrophage Polarization and Identification of Key Gene Modules in CESC. **(A)** Immune cell infiltration patterns in TCGA cervical cancer patients analyzed using the CIBERSORT algorithm. **(B)** Association between the M1/M2 macrophage ratio and overall survival. **(C)** Scale-free topology model fit analysis for selecting the optimal soft-thresholding power (β = 6, R² = 0.85). **(D)** Mean connectivity analysis under different soft-thresholding powers. **(E)** Correlation heatmap between gene modules and clinical traits.

Immune cell infiltration in the TCGA-CESC cohort was quantified using the CIBERSORT algorithm (**Figure 1A**). Higher M1 macrophage infiltration tended to be associated with improved survival, whereas higher M2 macrophage infiltration showed an opposite trend; however, neither association reached statistical significance (**Supplementary Figure S1A-B**, p > 0.05). In contrast, a reduced M1/M2 macrophage ratio was significantly associated with poorer patient prognosis (p < 0.05; **Figure 1B**). WGCNA was then performed to explore transcriptional programs associated with this prognostic phenotype, using dichotomized M1/M2 ratio groups (high vs. low) as the clinical trait (**Figures 1C, D**). Seven distinct co-expression modules were identified. Among these, the brown module exhibited the strongest positive correlation with the high M1/M2 ratio group (correlation = 0.25, p < 0.001) and was therefore prioritized for further analysis (**Figure 1E**). Univariate Cox regression analysis of genes within this module identified 121 genes significantly associated with overall survival (p < 0.05). Collectively, these findings indicate that the balance between M1- and M2-like macrophages, rather than their absolute abundance, is a critical prognostic factor in cervical cancer. Moreover, the identified gene module provides a biologically informed framework for subsequent molecular subtyping based on macrophage polarization-associated transcriptional programs.

### 2 Consensus clustering reveals two prognostic subtypes with distinct tumor microenvironment characteristics

**Figure 2:**
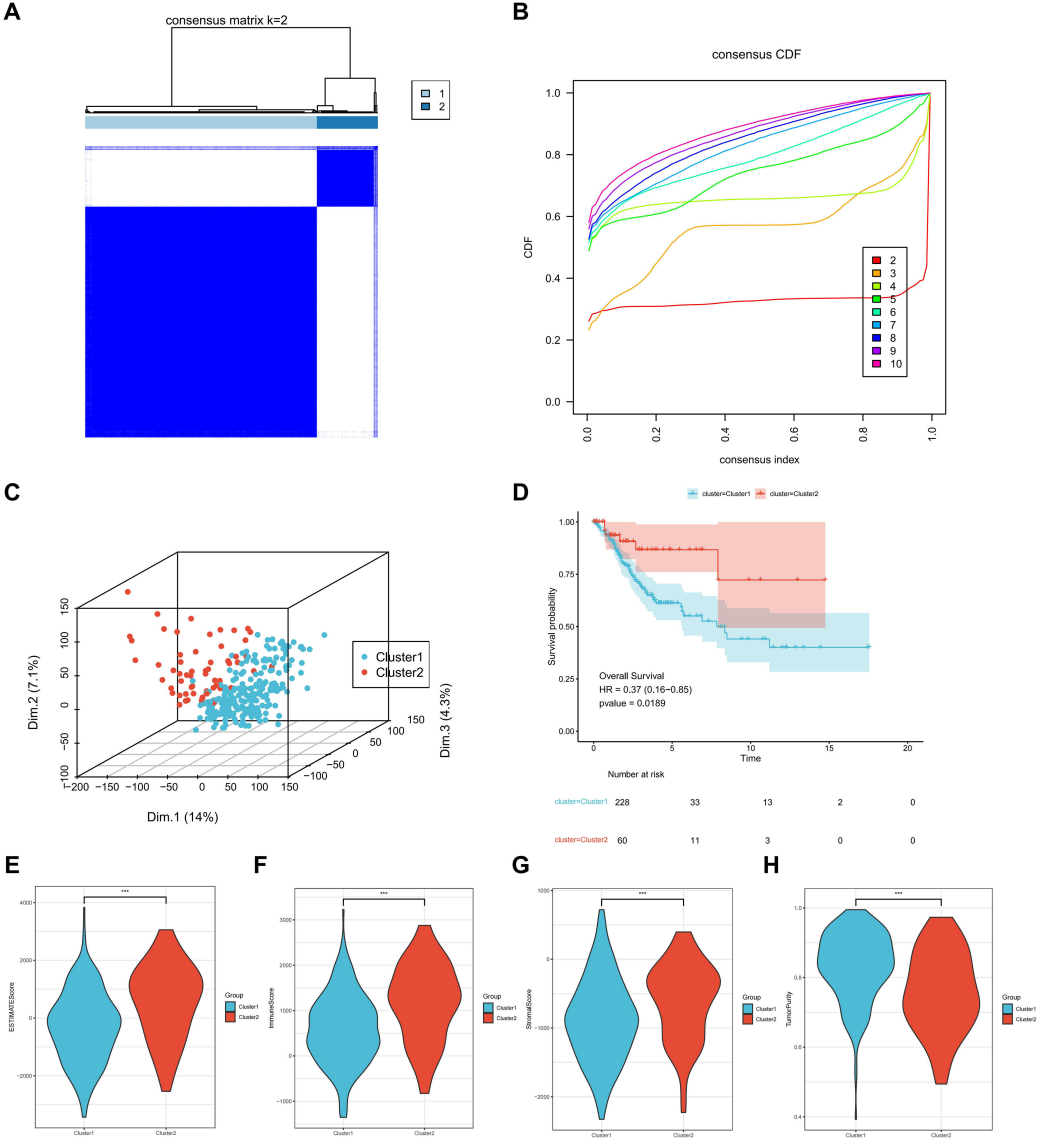
Consensus Clustering and Tumor Microenvironment Analysis. **(A)** Consensus clustering matrix when k = 2. **(B)** The consensus matrix and cumulative distribution function (CDF) plot confirm the stability of k = 2 clustering. **(C)** Principal component analysis (PCA) illustrates a clear separation between clusters 1 and 2. **(D)** Kaplan-Meier survival analysis of Cluster 1 and Cluster 2. **(E-H)** Comparison of ESTIMATE scores, immune scores, stromal scores, and tumor purity between the two clusters (*p<0.05; ***p<0.001).

Consensus clustering based on the expression profiles of the 121 macrophage polarization-associated prognostic genes delineated two robust molecular subtypes, designated C1 (n = 228) and C2 (n = 60) (**Figures 2A, B**). PCA confirmed clear transcriptomic separation between the two clusters (**Figure 2C**). Survival analysis demonstrated that patients in cluster C1 experienced significantly shorter overall survival compared with those in cluster C2 (**Figure 2D**). Evaluation of the tumor microenvironment using the ESTIMATE algorithm revealed that C1 tumors were characterized by significantly lower immune, stromal, and ESTIMATE scores, accompanied by higher tumor purity (**Figures 2E–H**). This profile is consistent with an immune-depleted or “cold” tumor phenotype. These results demonstrate that macrophage polarization-related transcriptional patterns define biologically and clinically distinct subtypes of cervical cancer, with unfavorable prognosis associated with a depleted immune- stromal landscape.

### 3 Functional characterization of differentially expressed genes between macrophage polarization-defined subtypes

**Figure 3:**
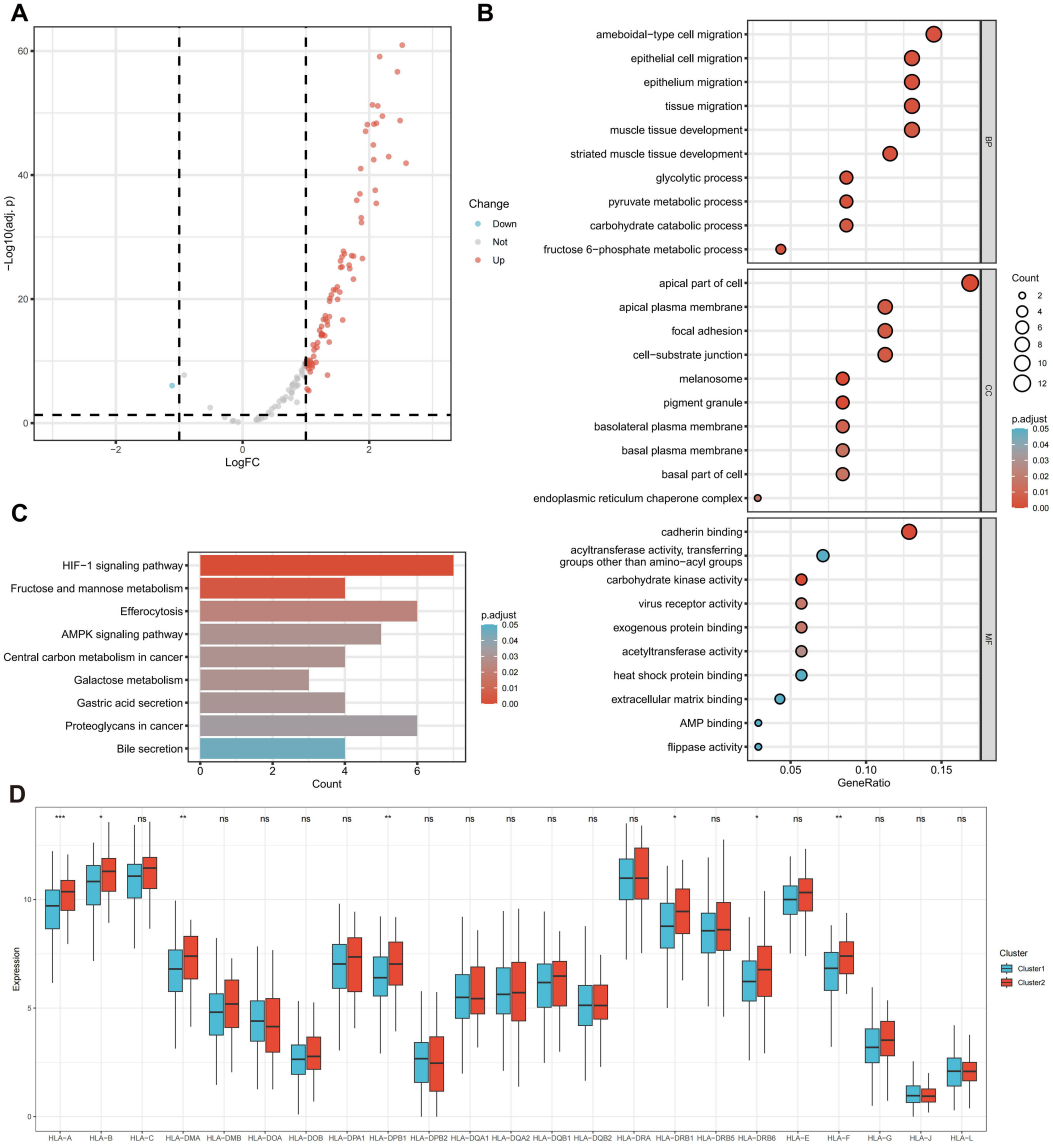
Analysis of differentially expressed genes (DEGs) and functional enrichment between clusters. **(A)** Volcano plot illustrating DEGs between the two clusters. **(B)** Gene Ontology (GO) enrichment analysis of DEGs. **(C)** Kyoto Encyclopedia of Genes and Genomes (KEGG) pathway analysis of DEGs. **(D)** Differential expression of HLA-related genes between the two clusters. *P<0.05, **P<0.01, ***P<0.001, ns, not significant.

Differential expression analysis identified 77 genes that significantly distinguished clusters C1 and C2 from the original 121-gene set (**Figure 3A**). Gene Ontology enrichment analysis revealed that these differentially expressed genes were primarily involved in cadherin binding, apical cellular localization, metabolic reprogramming-including fructose 6-phosphate metabolism and glycolysis-as well as tissue development and cell migration (**Figure 3B**). Consistently, Kyoto Encyclopedia of Genes and Genomes pathway analysis highlighted enrichment in metabolic pathways, such as central carbon metabolism, galactose metabolism, and bile secretion, as well as cancer-related pathways including proteoglycans in cancer and key signaling pathways such as hypoxia-inducible factor 1 and AMP-activated protein kinase signaling (**Figure 3C**). Notably, cluster C2 exhibited higher expression of multiple human leukocyte antigen genes, including HLA-A, HLA-B, HLA-DMA, and HLA-DPB1 (**Figure 3D**), indicating enhanced antigen presentation capacity and a more immunoreactive tumor microenvironment. Together, these results suggest that favorable prognosis is associated with coordinated metabolic remodeling and enhanced immune functionality, providing biological insight into macrophage polarization-associated clinical outcomes.

### 4 Multi-algorithm machine learning approach identifies core prognostic markers

**Figure 4:**
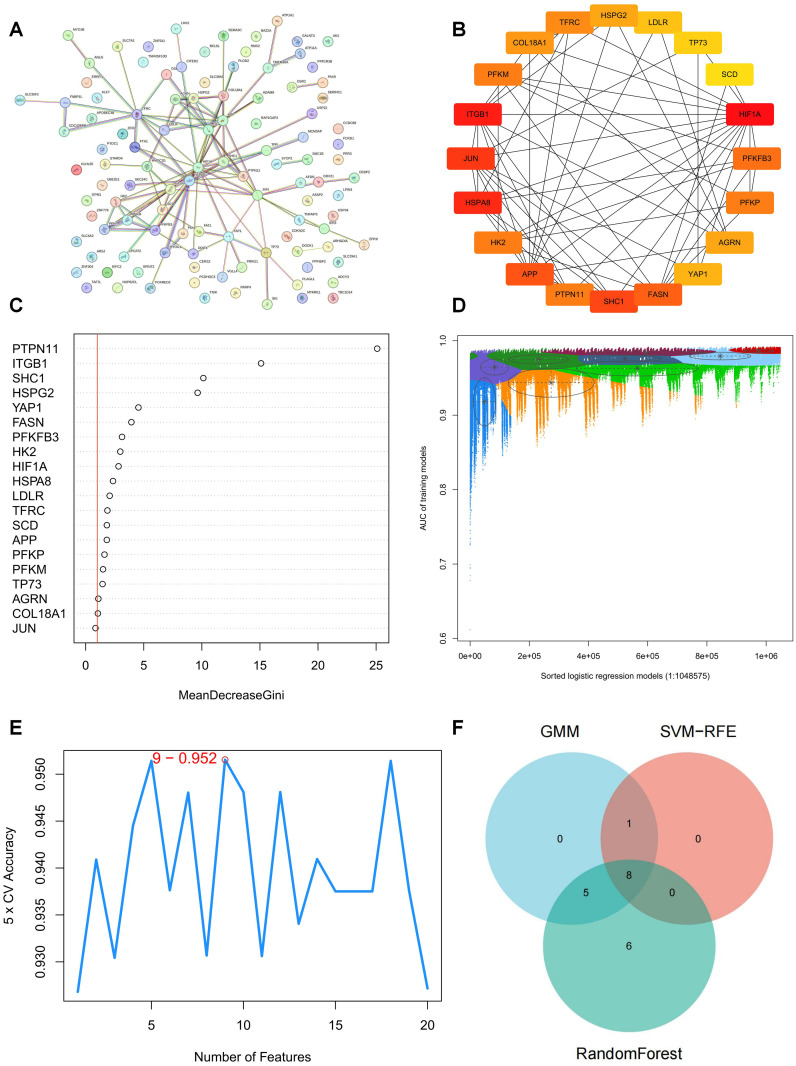
Screening and identification of prognostic markers through Protein-protein interaction (PPI) network and machine-learning approaches. **(A)** PPI network constructed using the STRING database. **(B)** Top 20 hub genes identified using the Maximal Clique Centrality (MCC) algorithm. **(C)** Random Forest analysis identifying genes with importance scores exceeding 1.0(n=19). **(D)** Gaussian Mixture Model (GMM) regression results (n=13). **(E)** Support Vector Machine-Recursive Feature Elimination (SVM-RFE) analysis (n=9). **(F)** Venn diagram showing the intersection of genes identified by three machine-learning methods.

To pinpoint central regulators, a PPI network was constructed from the DEGs (**Figure 4A**), and the top 20 hub genes were ranked using the MCC algorithm (**Figure 4B**). We then applied an integrated feature selection pipeline utilizing three distinct machine learning algorithms. Random Forest analysis identified 19 important genes (**Figures 4C, D**), GMM analysis selected 14 candidate genes (**Figure 4E**), and SVM-RFE identified an optimal subset of nine genes (**Figures 4F, G**). Intersection of the three approaches yielded eight high-confidence consensus genes-SHC1, PFKFB3, PTPN11, TFRC, HSPG2, LDLR, TP73, and SCD (**Figure 4H**)-which were considered core regulators and served as the basis for subsequent prognostic modeling.

### 5 Construction and validation of a macrophage polarization-related five-gene prognostic signature

**Figure 5:**
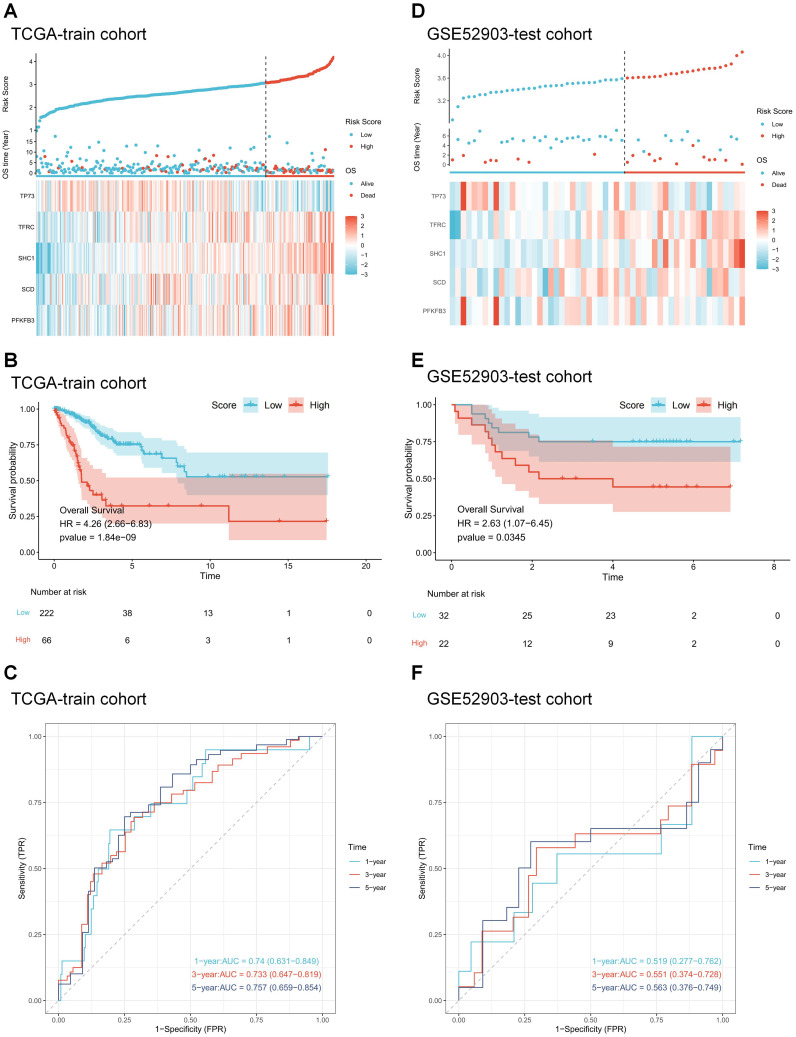
Construction and independent validation of the five-gene prognostic signature in cervical cancer. **(A, D)** Risk score distribution, patient survival status, and expression profiles of the five signature genes in the TCGA-train cohort **(A)** and GSE52903-test cohort **(D)**. The upper panels display the distribution of risk scores; the middle panels show the survival duration and status of individual patients; and the lower heatmaps illustrate the expression patterns of the five genes (TP73, TFRC, SHC1, SCD, and PFKFB3) across the low- and high-risk groups. Risk group assignment was based on the optimal cut-point determined by the surv_cutpoint function (survminer package). **(B, E)** Kaplan–Meier survival curves comparing the overall survival (OS) between high-risk and low-risk patients in the TCGA-train cohort **(B)** and GSE52903-test cohort **(E)**. Statistical significance was determined using the log-rank test. HR: hazard ratio; CI: confidence interval. **(C, F)** Time-dependent receiver operating characteristic (ROC) analysis evaluating the predictive accuracy of the five-gene signature at 1, 3, and 5 years in the TCGA-train cohort **(C)** and GSE52903-test cohort **(F)**. AUC, area under the curve; TPR, true positive rate; FPR, false positive rate.

LASSO Cox regression distilled the most informative prognostic features from the eight consensus genes while minimizing model complexity. Ten-fold cross-validation identified an optimal penalty parameter, resulting in a parsimonious five-gene prognostic signature comprising TP73, TFRC, SHC1, SCD, and PFKFB3 (**Supplementary Figure S2A-B**). A risk score was calculated for each patient in the TCGA-CESC cohort using the following formula: Risk Score = (0.3490 × SHC1) + (0.0356 × PFKFB3) + (0.2032 × TFRC) − (0.2659 × TP73) + (0.0095 × SCD). Patients were stratified into high- and low-risk groups according to the optimal cut-point determined using the surv_cutpoint function (survminer package v0.4.9) (**Figure 5A**). High-risk patients exhibited a markedly increased mortality rate and significantly shorter overall survival compared with low-risk patients (**Figure 5B**). Time-dependent ROC analysis demonstrated stable and satisfactory predictive performance at 1, 3, and 5 years (**Figure 5C**).

**Figure 6:**
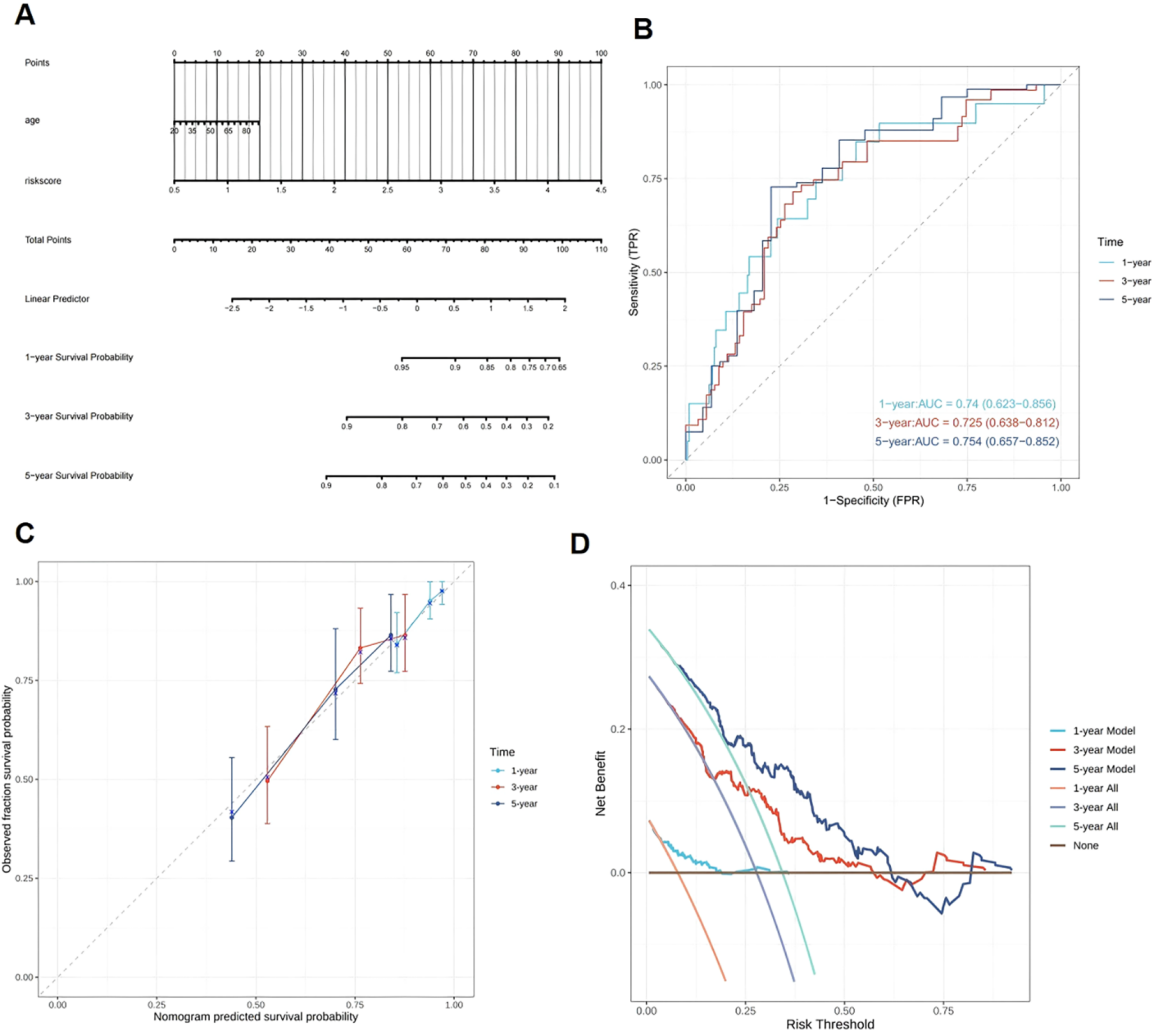
Development and systematic evaluation of a prognostic nomogram for patients with cervical cancer. **(A)** Nomogram integrating patient age and the risk score to estimate 1-, 3-, and 5-year overall survival probabilities in the TCGA_CESC. **(B)** ROC curves evaluating the predictive performance of the prognostic nomogram at 1-, 3-, and 5-year time points in the TCGA_CESC. **(C)** Calibration curves assessing the agreement between predicted and observed overall survival probabilities at 1, 3, and 5 years. **(D)** DCA evaluating the potential clinical net benefit of the nomogram across a range of threshold probabilities at 1-, 3-, and 5-year overall survival.

The prognostic value of the five-gene signature was independently validated in the GSE52903 cohort (**Figure 5D**), in which high-risk patients similarly showed significantly poorer survival outcomes and statistically significant prognostic stratification; however, the time-dependent AUC values were substantially attenuated (1-year AUC = 0.519, 3-year AUC = 0.551, 5-year AUC = 0.563), indicating limited discriminatory accuracy in this external cohort (**Figures 5E, F**). For clinical application, a prognostic nomogram integrating the five-gene risk score and patient age was constructed to predict 1-, 3-, and 5-year overall survival (**Figure 6A**), and its predictive accuracy, calibration, and clinical net benefit were systematically evaluated (**Figures 6B–D**). The proportional hazards assumption was confirmed for the five-gene model (global Schoenfeld test: χ² = 7.45, df = 5, p = 0.19; **Supplementary Figure S3**).

### 6 Risk score stratifies immune infiltration patterns and predicts reduced chemosensitivity

**Figure 7:**
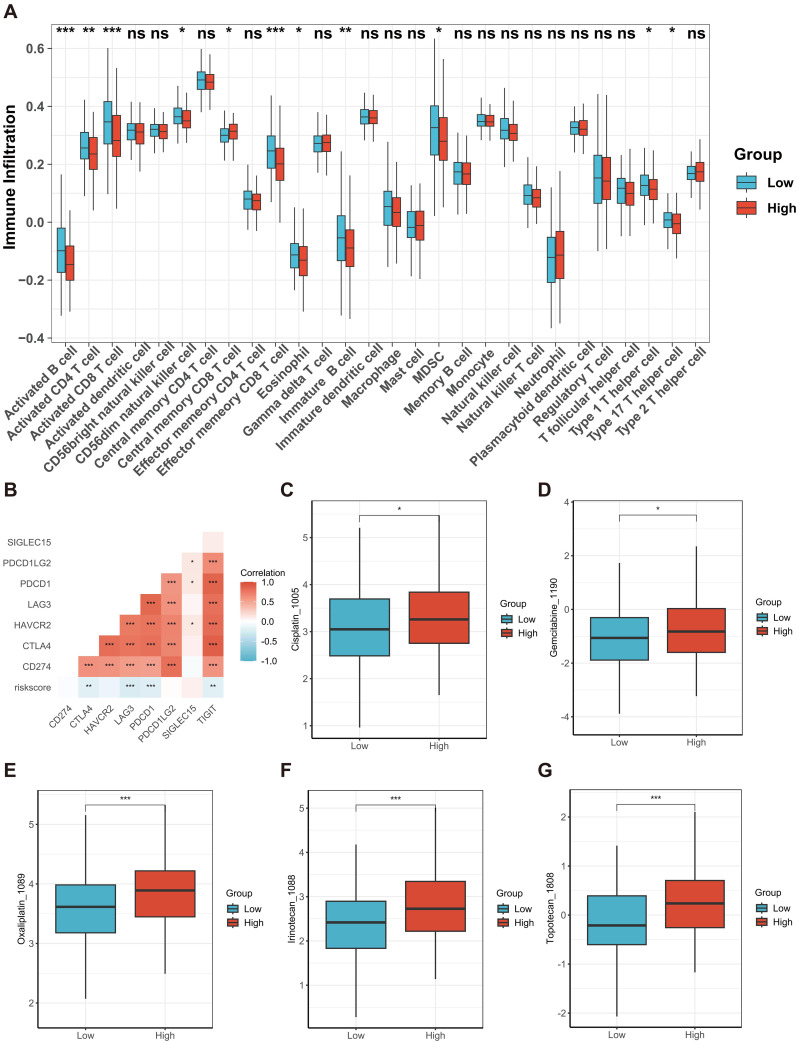
The prognostic risk score stratifies distinct immune landscapes and predicts chemotherapeutic response. **(A)** Comparison of immune cell infiltration levels between the high- and low-risk groups, as quantified by single-sample gene set enrichment analysis (ssGSEA). The infiltration scores of 25 immune cell subsets are shown. Significance was determined by the Wilcoxon rank-sum test (p < 0.05). **(B)** Correlation heatmap depicting the association between the risk score and the expression levels of key immune checkpoint molecules. Pearson correlation coefficients and their statistical significance are indicated. **(C-G)** Comparison of the predicted half-maximal inhibitory concentration (IC_50_) values for commonly used chemotherapeutic agents (C: cisplatin, D: gemcitabine, E: oxaliplatin, F: irinotecan, G: topotecan) between the high- and low-risk groups. Wilcoxon rank-sum test was used for statistical comparison; *P<0.05, **P<0.01, ***P<0.001, ns, not significant.

Immune infiltration, immune checkpoint expression, and predicted drug sensitivity were systematically compared between risk groups to characterize the immunobiological features associated with the five-gene prognostic signature. ssGSEA-based immune infiltration analysis revealed a globally suppressed antitumor immune landscape in the high-risk group. Infiltration levels of multiple effector immune populations-including activated B cells, activated CD4^+^ and CD8^+^ T cells, CD56^dim natural killer cells, effector memory CD8^+^ T cells, and type 1 and type 17 T helper cells-were significantly reduced. Eosinophils, immature B cells, and myeloid-derived suppressor cells were also decreased, whereas central memory CD8^+^ T cells were significantly increased in the high-risk group (**Figure 7A**). Correlation analysis demonstrated significant negative associations between the risk score and key inhibitory immune checkpoints, including CTLA4, LAG3, PDCD1 (PD-1), and TIGIT (p < 0.05). Negative trends were observed for CD274 (PD-L1) and HAVCR2 (TIM-3), although these did not reach statistical significance. In contrast, weak positive correlations were observed for PDCD1LG2 (PD-L2) and SIGLEC15 (**Figure 7B**).

Drug sensitivity prediction based on the GDSC database indicated that high-risk patients exhibited significantly higher predicted IC_50_ values for several chemotherapeutic agents commonly recommended in the NCCN guidelines for cervical cancer management, including cisplatin, oxaliplatin, gemcitabine, topotecan, and irinotecan, suggesting reduced chemosensitivity(**Figures 7C–G**). In contrast, no significant differences were observed for 5-fluorouracil or paclitaxel between the two risk groups (**Supplementary Figure S4A-B**). Taken together, these results indicate that the macrophage polarization-related risk score delineates an immunologically inert or “cold” tumor phenotype characterized by attenuated effector immune infiltration, limited immune checkpoint engagement, accompanied by reduced sensitivity to multiple commonly utilized chemotherapeutic agents in cervical cancer management. This signature captures a clinically relevant axis of tumor-immune interaction and therapeutic vulnerability, providing a biologically informed basis for subsequent functional validation.

### 7 Single-cell transcriptomic profiling identifies tumor-intrinsic TFRC as a key driver of M2 macrophage polarization

**Figure 8:**
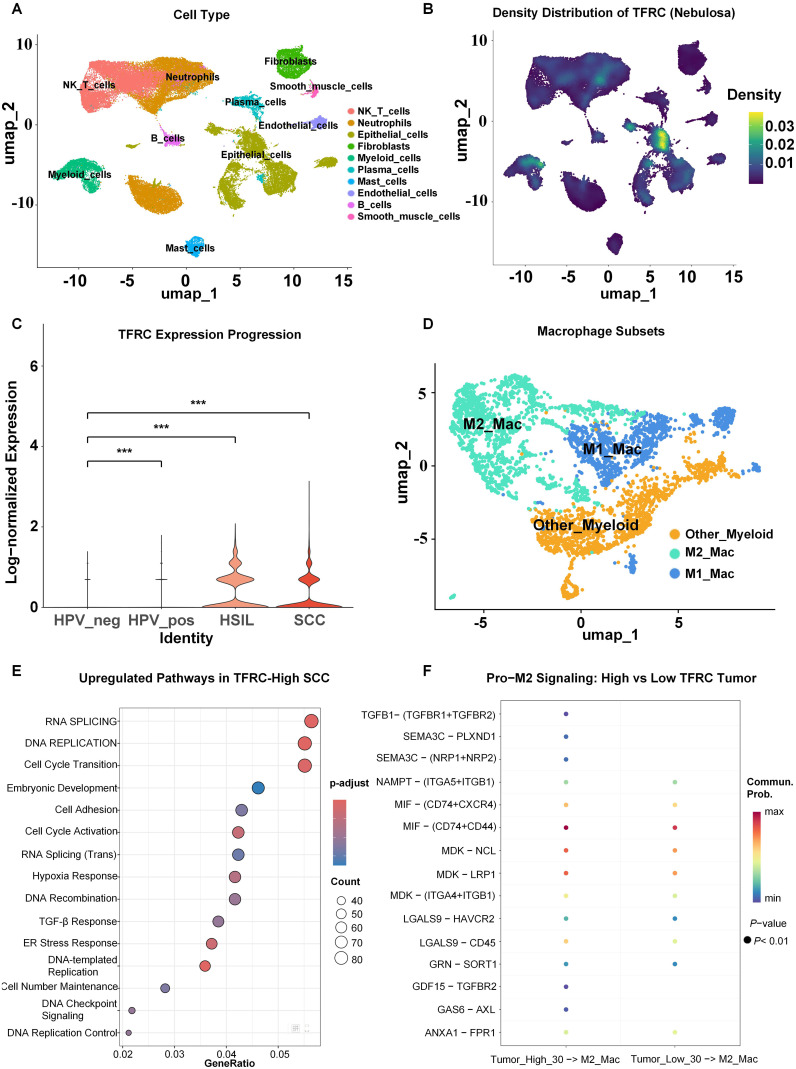
Single-cell transcriptomic profiling identifies tumor-intrinsic TFRC as a key driver of M2 macrophage polarization. **(A)** UMAP plot illustrating the global single-cell transcriptomic landscape of the cervical cancer microenvironment (GSE208653), identifying ten major cell lineages including epithelial cells, myeloid cells, T/NK cells, and fibroblasts. **(B)** Nebulosa-based expression density plot revealing the tumor-intrinsic enrichment of TFRC, showing predominant and specific expression within the malignant epithelial cell population compared to immune or stromal compartments. **(C)** Violin plot demonstrating the progressive upregulation of TFRC expression along the disease continuum, peaking in invasive squamous cell carcinoma (SCC) (***P<0.001). **(D)** UMAP visualization of isolated myeloid populations, with macrophage subsets definitively annotated into M1 and M2 phenotypes. **(E)** Gene Ontology **(GO)** enrichment analysis highlighting upregulated biological processes in TFRC-High SCC cells, including hyper-proliferative signatures, metabolic stress responses (hypoxia and ER stress), and TGF-β signaling. **(F)** Bubble plot of CellChat-inferred ligand-receptor interactions from TFRC-High and TFRC-Low tumor subgroups toward M2 macrophages, showing enhanced pro-M2 signaling via the TGF-β, MIF, and MDK axes.

To extend our bulk-scale observations to cellular resolution, we characterized the single-cell transcriptomic landscape of cervical cancer (GSE208653). This approach delineated ten major cell lineages within the microenvironment, with unsupervised clustering and marker annotation revealing distinct populations of epithelial cells, myeloid cells, T/NK cells, and fibroblasts (**Figure 8A**).

Crucially, we evaluated the continuous expression density of the five modeled genes across this landscape. Among the genes with positive coefficients in our LASSO regression model (indicating a risk-promoting role), TFRC stood out uniquely. While other risk genes exhibited either dispersed or stroma-biased expression patterns (**Supplementary Figure S5**), TFRC was predominantly and specifically enriched within the malignant epithelial cell (SCC) population, with minimal expression in immune or stromal compartments (**Figure 8B**). This striking contrast firmly established TFRC as a predominantly tumor-intrinsic risk factor. Furthermore, violin plot analysis demonstrated a progressive and significant upregulation of TFRC expression along the disease continuum, advancing from HPV-negative normal tissues to HSIL and peaking in invasive SCC (**Figure 8C**, P < 0.001).

Given the prominent role of macrophages in the cervical cancer microenvironment, we next investigated the downstream communicative effects of tumor-intrinsic TFRC. Macrophage populations were definitively annotated into M1 and M2 phenotypes using AUCell (**Figure 8D**). To elucidate the functional landscape of cells driving this microenvironment, malignant epithelial cells were stratified into TFRC-High and TFRC-Low subgroups. Gene Ontology (GO) enrichment analysis revealed that TFRC-High SCC cells were characterized by hyper-proliferative signatures (DNA replication and cell cycle transition) and profound metabolic stress, including response to oxygen levels (hypoxia) and endoplasmic reticulum stress (**Figure 8E**). Notably, these malignant clones also exhibited significant enrichment in the TGF-β signaling response, indicating the activation of a potent TGF-β autocrine/paracrine axis that potentially drives immunosuppressive microenvironmental remodeling (**Figure 8E**).

Finally, intercellular communication analysis via CellChat inferred a computationally predicted ligand-receptor interaction network associated with TFRC expression. Compared to the TFRC-Low subgroup, TFRC-High malignant cells exhibited significantly enhanced pro-M2 signaling, predicted to exhibit enhanced paracrine crosstalk enriched for secreted ligand-receptor pathways, including the TGF-β, MIF, and MDK axes (**Figure 8F**). These computational findings suggest that tumor-intrinsic TFRC may promote an immunosuppressive niche through M2 macrophage polarization, providing a computational rationale for its selection for *in vitro* validation.

### 8 TFRC is functionally associated with M2-like polarization of TAMs

**Figure 9:**
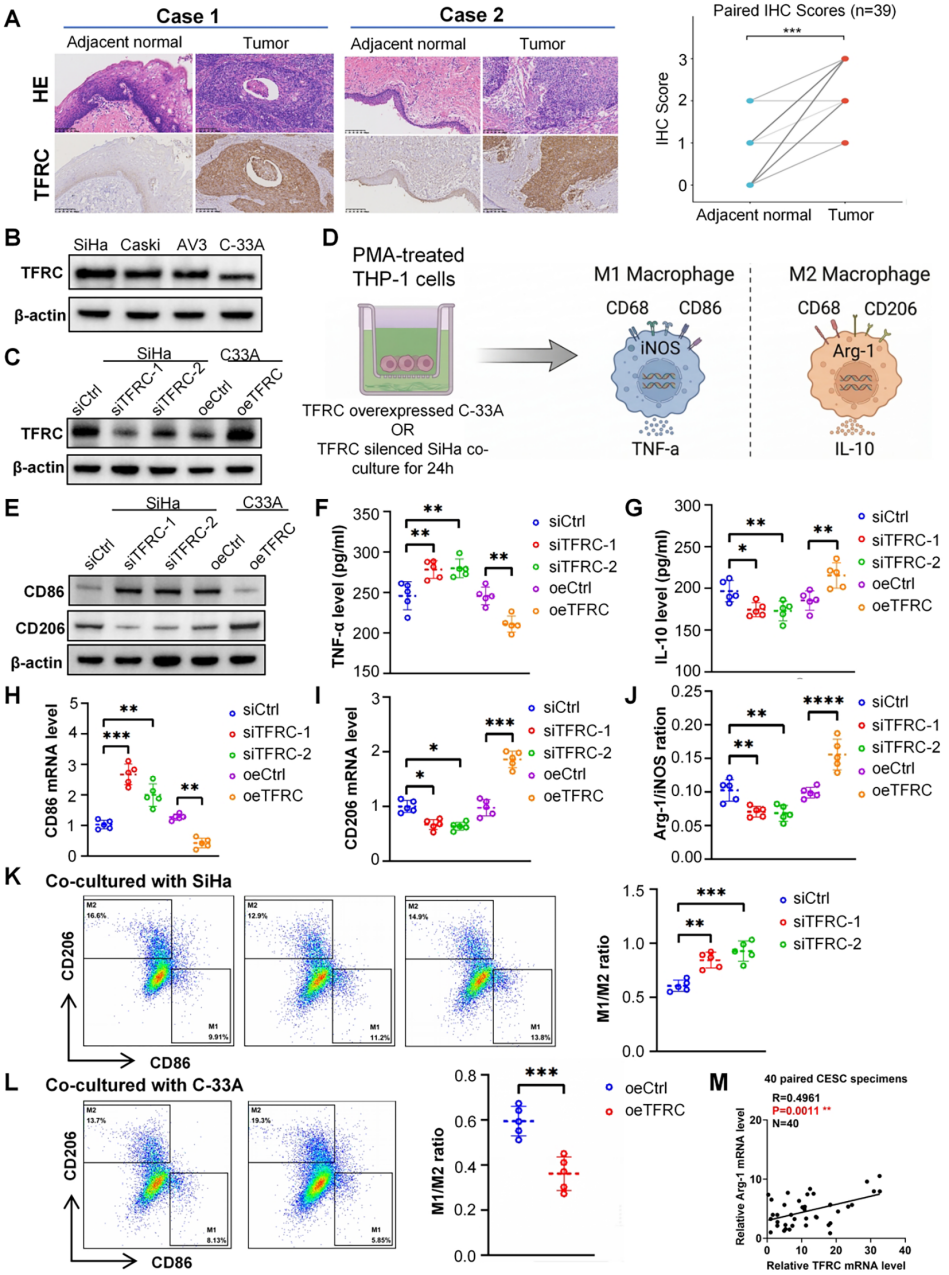
Functional validation of tumor-intrinsic TFRC in orchestrating M2-like macrophage polarization. **(A)** Representative IHC and H&E staining of TFRC in paired cervical cancer and adjacent normal tissues (Case 1 and Case 2). The right panel shows the IHC scores for 39 paired specimens (Wilcoxon signed-rank test, ***P < 0.001). **(B)** Baseline TFRC protein expression in cervical cancer cell lines (SiHa, CaSki, AV3, and C-33A). **(C)** Western blot confirmation of TFRC silencing in SiHa cells and overexpression in C-33A cells. **(D)** Schematic illustration of the co-culture system using PMA-differentiated THP-1 macrophages. **(E)** Western blot analysis of macrophage polarization markers CD86 and CD206 in THP-1-derived macrophages following co-culture with TFRC-overexpressing C-33A cells or TFRC-silenced SiHa cells. **(F-G)** ELISA quantification of secreted TNF-α and IL-10 levels in macrophage supernatants (Student’s t-test, **P < 0.01). **(H-J)** RT-qPCR analysis of mRNA levels for CD86, CD206, and the iNOS/Arg-1 ratio in macrophages (Student’s t-test, * *P*<0.05, ** *P*<0.01, **P<0.001, ****P<0.0001). **(K)** Representative flow cytometry dot plots (x-axis: CD86; y-axis: CD206) of macrophages co-cultured with TFRC-silenced SiHa cells (siCtrl, siTFRC-1, siTFRC-2), and corresponding M1/M2 ratio quantification. **(L)** Representative flow cytometry dot plots of macrophages co-cultured with TFRC-overexpressing C-33A cells (oeCtrl, oeTFRC), and corresponding M1/M2 ratio quantification. Data are presented as mean ± SD from three independent experiments. **(M)** Pearson correlation analysis between TFRC and Arg-1 mRNA expression levels in 40 CESC tumor specimens as determined by RT-qPCR (Pearson’s r = 0.4961, P = 0.0011).

Following our in silico findings, we first validated TFRC protein expression via immunohistochemical (IHC) analysis in our clinical cohort (n=39). Consistent with the scRNA-seq data, tumor tissues exhibited significantly higher TFRC protein levels compared to adjacent non-malignant counterparts (**Figure 9A**, *P* < 0.001). These protein-level observations, coupled with the substantial contribution of TFRC to our prognostic model, solidified its selection for subsequent functional interrogation.

To establish appropriate experimental models, we screened TFRC expression across multiple cervical cancer cell lines, identifying the highest endogenous levels in SiHa cells and the lowest in C-33A cells (**Figure 9B**). We subsequently performed TFRC silencing (siTFRC-1/2) in SiHa cells and overexpression (oeTFRC) in C-33A cells, with efficiency confirmed by Western blot (**Figure 9C**).

To investigate the paracrine influence of tumor-intrinsic TFRC on macrophage plasticity, we utilized a co-culture system with PMA-differentiated THP-1 macrophages (**Figure 9D**). At the protein level, Western blot analysis demonstrated that TFRC knockdown in SiHa cells increased the expression of the M1 marker CD86, whereas TFRC overexpression in C-33A cells significantly boosted the M2 marker CD206 (**Figure 9E**). Functional changes in the secretory profile were assessed via ELISA, which revealed that TFRC deficiency promoted the release of the pro-inflammatory cytokine TNF-α (**Figure 9F**), while TFRC overexpression stimulated the production of the anti-inflammatory cytokine IL-10 (**Figure 9G**).

Consistent with these findings, RT-qPCR analysis showed that TFRC knockdown upregulated CD86 mRNA (**Figure 9H**), whereas TFRC overexpression enhanced CD206 levels (**Figure 9I**). Notably, the Arg-1/iNOS mRNA ratio—a key indicator of M2-skewed polarization—was significantly decreased following TFRC silencing and increased upon TFRC overexpression (**Figure 9J**). Flow cytometric analysis further quantified these phenotypic shifts; in SiHa co-cultures, TFRC silencing significantly expanded the M1-like (CD86^+^) macrophages, leading to an elevated M1/M2 ratio (**Figure 9K**). Conversely, exposure to TFRC-overexpressing C-33A cells induced a robust M2-like transition, characterized by a markedly decreased M1/M2 ratio (**Figure 9L**).In clinical specimens from 40 cervical cancer patients, TFRC mRNA level was significantly positively correlated with Arg-1 expression as determined by RT-qPCR (**Figure 9M**).

**Figure 10:**
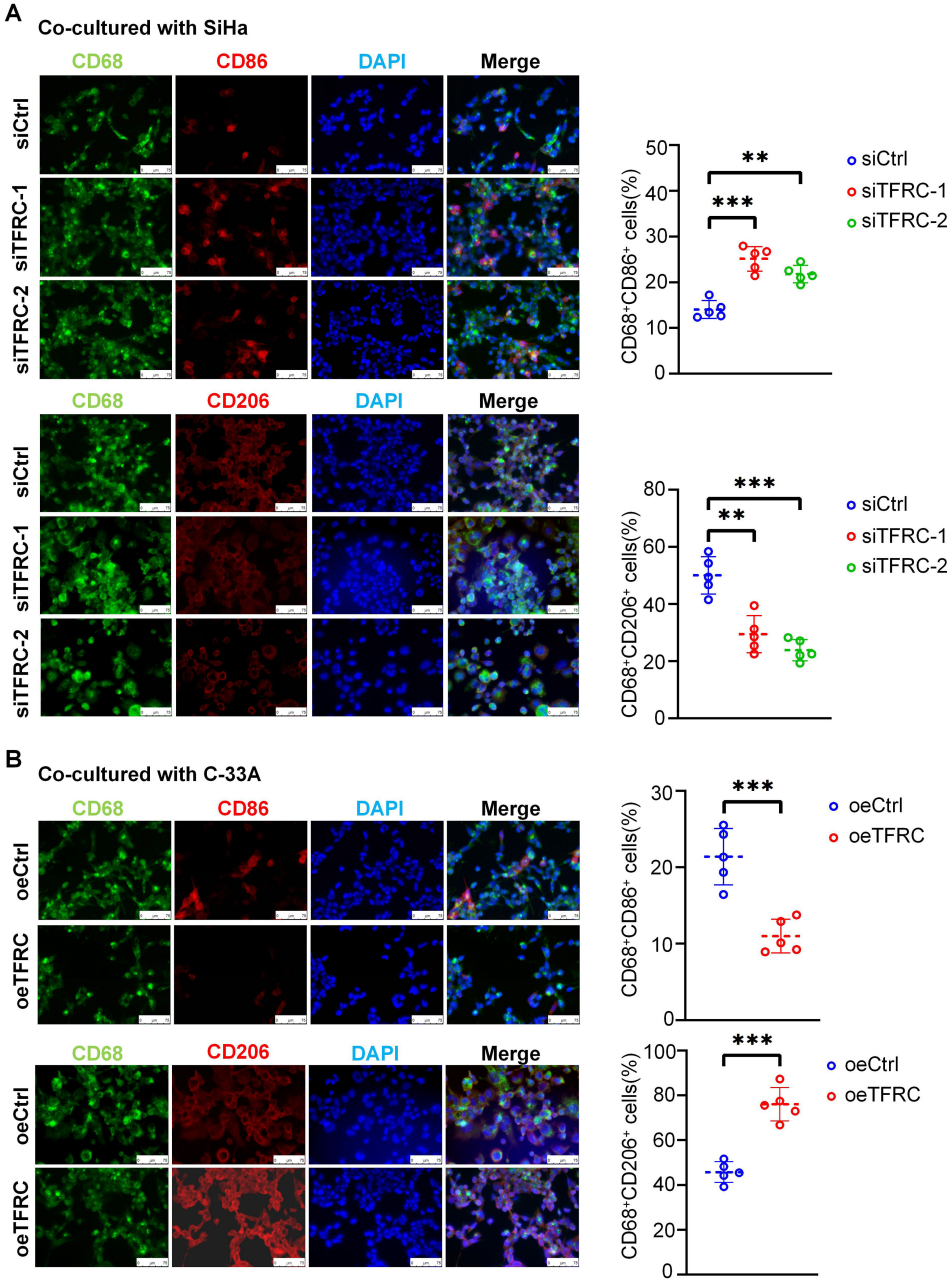
Multiplex immunofluorescence (mIF) validates TFRC-mediated regulation of macrophage polarization. mIF images and corresponding quantitative analysis of M1-like (CD68^+^CD86^+^) and M2-like (CD68^+^CD206^+^) macrophage populations following co-culture with TFRC-silenced SiHa cells **(A)** and TFRC-overexpressing C-33A cells **(B)**. Student’s t-test, **P<0.01, ***P<0.001. Data are presented as mean ± SD from three independent experiments.

Finally, multiplex immunofluorescence (mIF) provided spatial and phenotypic confirmation of this polarization axis (**Figure 10**). The proportion of CD68^+^CD86^+^ M1-like macrophages was significantly increased in the TFRC-silenced SiHa co-culture group (**Figure 10A**). In contrast, TFRC overexpression in C-33A cells led to a substantial expansion of the CD68^+^CD206^+^ M2-like macrophages (**Figure 10B**). Collectively, these results provide convergent phenotypic and functional evidence that tumor-derived TFRC acts as a potent orchestrator of M2-like macrophage polarization in the cervical cancer microenvironment.

## Discussion

Moving beyond traditional metrics of immune cell abundance, our study establishes the functional plasticity of TAMs as the primary axis for prognostic stratification. By integrating bulk and single-cell transcriptomic profiling with a multi-algorithm machine learning framework, we systematically delineated macrophage polarization-associated molecular features and developed a robust prognostic gene signature. Crucially, we experimentally validated the role of a key signature gene, TFRC, in orchestrating an immunosuppressive M2-like macrophage phenotype. Collectively, these findings establish an immune-metabolic biomarker framework for prognostic stratification and provide biological insights into how tumor-intrinsic iron metabolism shapes the tumor microenvironment.

Our analyses confirmed the prognostic relevance of macrophage polarization balance in cervical cancer. A reduced M1/M2 macrophage ratio was significantly associated with inferior overall survival, supporting the concept that the functional phenotype of TAMs more accurately reflects the immunological status of the tumor than macrophage abundance alone (38–40). Consensus clustering based on macrophage polarization-associated prognostic genes identified two distinct molecular subtypes. Tumors in cluster C1 exhibited significantly worse survival, lower immune and stromal scores, and higher tumor purity, consistent with an immune-depleted phenotype (41). In contrast, C2 tumors displayed an immunologically engaged microenvironment, indicating that macrophage polarization patterns are closely linked to global differences in the immune contexture (42).

At the transcriptomic level, C1 tumors were characterized by metabolic reprogramming—including glycolysis, HIF-1 signaling, and AMPK signaling—pathways known to influence both tumor adaptation and immune function (43–48). Notably, cluster C2 exhibited higher expression of multiple HLA class I and II genes, indicating enhanced antigen presentation capacity. In contrast, reduced HLA expression in C1 contributes to impaired immune recognition and immune evasion, providing a plausible molecular basis for the observed prognostic differences between the subtypes (49, 50).

To translate these biological insights into a clinically applicable tool, we constructed and validated a concise five-gene prognostic signature (TP73, TFRC, SHC1, SCD, and PFKFB3). The constituent genes reflect interconnected processes involving signal transduction (51), metabolic regulation (52–54), and cellular stress responses (55), reinforcing the biological plausibility of the model. Beyond risk stratification, pharmacogenomic prediction revealed that high-risk patients exhibited higher predicted IC_50_ values for multiple NCCN-recommended chemotherapeutic agents, including platinum-based compounds and topoisomerase inhibitors. Rather than acting as direct predictors of drug-specific resistance, this reduced predicted sensitivity reflects an underlying “immunologically inert” and metabolically reprogrammed tumor phenotype that is fundamentally less permissive to effective cytotoxic therapy.

A distinctive strength of this study lies in the use of single-cell transcriptomic analysis (GSE208653) to bridge the gap between bulk-level risk stratification and cellular-level mechanism. While bulk transcriptomics often masks the specific cellular source of prognostic signals, our scRNA-seq profiling allowed for a precise cellular localization of all five signature genes. TFRC emerged as the most biologically relevant candidate, uniquely and predominantly enriched within the malignant epithelial compartment rather than the stroma. The progressive upregulation of TFRC from normal tissue to HSIL and invasive SCC (*P* < 0.001) further highlights its role as a metabolic driver of the malignant continuum.

Mechanistically, our study elucidates how tumor-intrinsic metabolic stress translates into extrinsic immune suppression. GO enrichment analysis revealed that TFRC-high SCC cells are characterized by intense hypoxia and endoplasmic reticulum (ER) stress. These metabolic perturbations are known to drive a pro-tumorigenic “secretory” phenotype. Accordingly, intercellular communication analysis via CellChat computationally predicted a paracrine crosstalk originating from TFRC-high tumors toward M2 macrophages, primarily mediated by the TGF-β, MIF, and MDK signaling axes. This predicted ligand-receptor interactome suggests that TFRC does not function in isolation but acts as a nodal orchestrator of a self-reinforcing immunosuppressive loop.

To bridge these high-resolution in silico predictions with clinical and functional evidence, we first validated TFRC protein expression in our paired patient cohort (n=39). Consistent with the single-cell findings, IHC analysis confirmed a significant tumor-specific enrichment of TFRC, primarily characterized by moderate-to-strong cytoplasmic staining in malignant regions. This robust clinical validation, coupled with TFRC’s established role in cellular iron uptake and its substantial weight in our prognostic model, solidified its selection as the lead candidate for functional interrogation of the tumor-macrophage axis.TFRC has been widely reported to be overexpressed across diverse malignancies and is frequently associated with enhanced proliferative capacity, metabolic adaptation, and unfavorable clinical outcomes, underscoring its importance as a core component of tumor iron metabolism. In addition, iron metabolism has emerged as a key determinant of macrophage functional states, with iron availability influencing the balance between pro-inflammatory and immunosuppressive phenotypes (56). It should be noted, however, that the relationship between iron and macrophage polarization is not unidirectional. Several studies have reported that iron overload promotes pro-inflammatory M1 macrophage activation, while iron restriction has been associated with suppressed M1 responses in certain inflammatory contexts (57, 58). Notably, a parallel mechanism was demonstrated in hepatocellular carcinoma, where tumor cells overexpressing TFRC competitively sequester iron from macrophages, creating an iron-depleted microenvironment that drives TAM polarization toward an immunosuppressive M2 phenotype via HIF-1α activation (59). These observations collectively suggest that the pro-M2 effect of TFRC-driven iron metabolism is context-dependent and likely reflects tumor-specific iron redistribution rather than systemic iron overload, underscoring the need for future studies to delineate the precise downstream mediators of TFRC-mediated immune remodeling in gynecological malignancies.

Within this biological context, our co-culture experiments demonstrate that tumor-derived TFRC actively shapes the macrophage functional landscape. By driving the M2-like skewed polarization of macrophages and the subsequent secretion of inhibitory cytokines such as IL-10, tumor-intrinsic TFRC effectively remodels a specialized immunosuppressive niche, thereby facilitating immune evasion within the cervical cancer microenvironment. These findings refine the interpretation of the prognostic model by distinguishing between its system-level and mechanistic dimensions. While the risk score integrates coordinated transcriptional programs associated with macrophage polarization, TFRC serves as an experimentally tractable node that provides functional evidence linking tumor-intrinsic metabolic regulation to immune cell behavior. This distinction underscores that the prognostic signature is not a black-box classifier but reflects biologically interpretable tumor-immune interactions.

The association between TFRC expression, macrophage polarization, and predicted therapeutic sensitivity warrants careful interpretation. Our findings do not suggest that TFRC functions as a direct drug target for the agents evaluated, nor do they imply that TFRC expression alone determines treatment response. Rather, TFRC may represent a nodal component within a broader metabolic-immune regulatory axis that shapes the tumor microenvironment and indirectly influences therapeutic vulnerability. To build upon these findings, ongoing investigations in our laboratory are actively dissecting the downstream secretome alterations induced by iron flux to clarify how TFRC-driven tumor metabolism interfaces with T-cell exclusion and functional exhaustion.

Several limitations of this study should be acknowledged. First, the prognostic model was developed and validated using retrospective transcriptomic datasets, and prospective clinical validation will be necessary. Second, immune-related therapeutic implications were inferred indirectly, as large, well-annotated cervical cancer immunotherapy cohorts remain limited. Third, although TFRC was functionally linked to macrophage polarization, the precise downstream molecular mediators of this interaction remain to be fully elucidated. Fourth, although the five-gene signature retained statistically significant prognostic stratification in the external GSE52903 cohort, the time-dependent AUC values were substantially attenuated compared with the training cohort (1-year: 0.519; 3-year: 0.551; 5-year: 0.563). This attenuation may partly reflect the limited sample size of the external cohort (n = 55), as well as systematic platform differences between TCGA RNA-seq and GEO microarray data, and underscores the need for further validation in larger, prospective, platform-matched cohorts. These limitations define important avenues for future investigation rather than detract from the central conclusions regarding the TFRC-mediated metabolic-immune axis.

## Conclusion

In summary, this study establishes a cross-scale, macrophage polarization-centered prognostic framework that captures clinically relevant immune-metabolic heterogeneity in cervical cancer. The five-gene signature provides robust prognostic stratification and predicts therapeutic vulnerability with high biological interpretability. Furthermore, single-cell resolution and functional validation of TFRC reveal a direct mechanistic link between tumor-intrinsic iron metabolism and the immunosuppressive M2-like niche. Together, these findings offer a biologically grounded perspective on tumor-immune interactions, providing a robust foundation for future translational strategies aimed at targeting immune-metabolic vulnerabilities to overcome therapeutic resistance in cervical cancer.

## Data Availability

The datasets presented in this study can be found in online repositories. The names of the repository/repositories and accession number(s) can be found in the article/supplementary material.
