# Supplementary material for: Cross-scale modeling reveals a TFRC-driven immunosuppressive macrophage niche in cervical cancer
Source: Front Immunol. 2026 Jul 17;17:1872944. doi: 10.3389/fimmu.2026.1872944 (PMC13423990; doi:10.3389/fimmu.2026.1872944)

Supplementary Figure S1

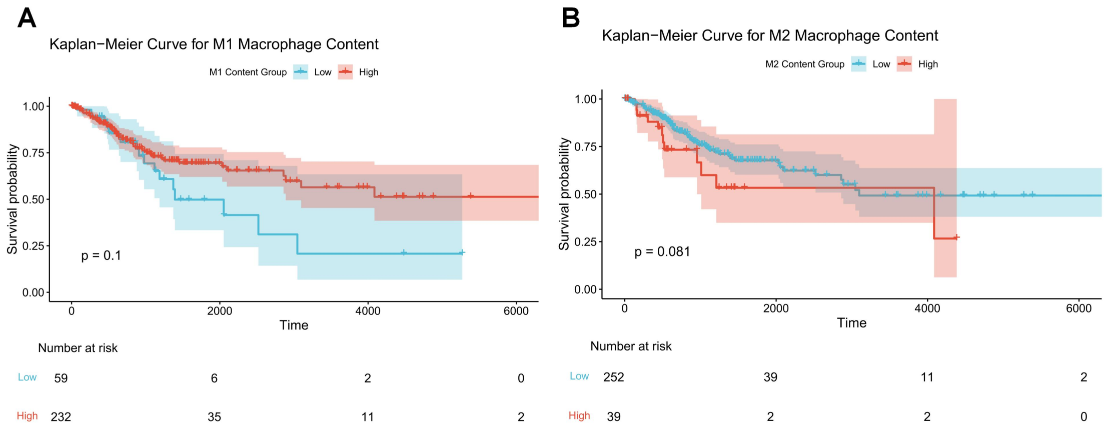

Supplementary Figure S1. Association between macrophage infiltration and overall survival in cervical cancer. (A) Kaplan – Meier survival curves comparing overall survival between patients with high and low M1 macrophage infiltration. (B) Kaplan – Meier survival curves comparing overall survival between patients with high and low M2 macrophage infiltration.

Supplementary Figure S2

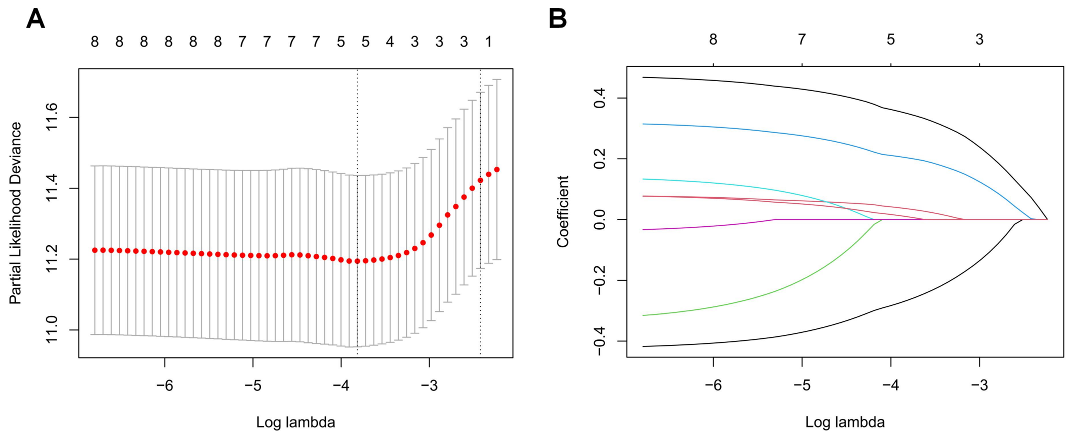

Supplementary Figure S2. Feature selection and parameter optimization using the LASSO regression model. (A)LASSO coefficient profiles of the eight candidate genes. (B) Selection of the optimal penalization coefficient (λ) in the LASSO model via 10-fold cross-validation.

Supplementary Figure S3

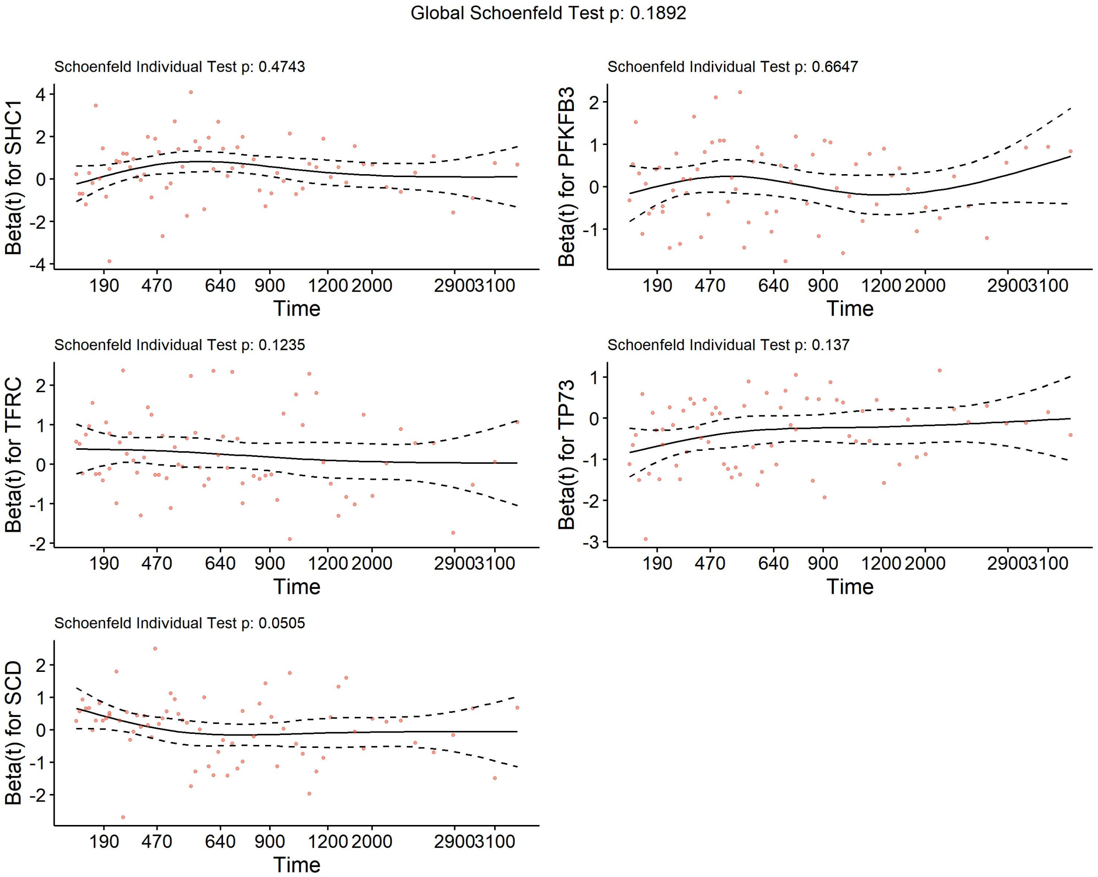

Supplementary Figure S3. Schoenfeld residual plots for each component of the five-gene multivariate Cox model. Scaled Schoenfeld residuals are plotted against follow-up time for each of the five signature genes. The absence of significant time-dependent trends confirms that the proportional hazards assumption is satisfied for the model (global Schoenfeld test: $\chi^2 = 7.45$, df = 5, $P = 0.19$).

Supplementary Figure S4

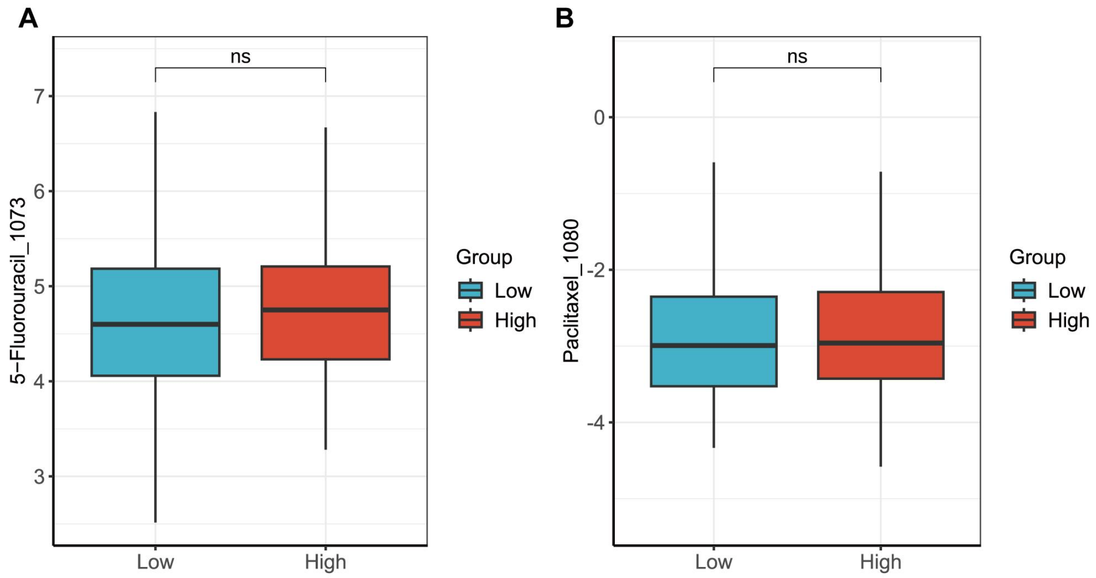

Supplementary Figure S4. Comparison of the predicted half-maximal inhibitory concentration (IC50) values for commonly used chemotherapeutic agents. (A) 5-fluorouracil (B) paclitaxel

Supplementary Figure S5

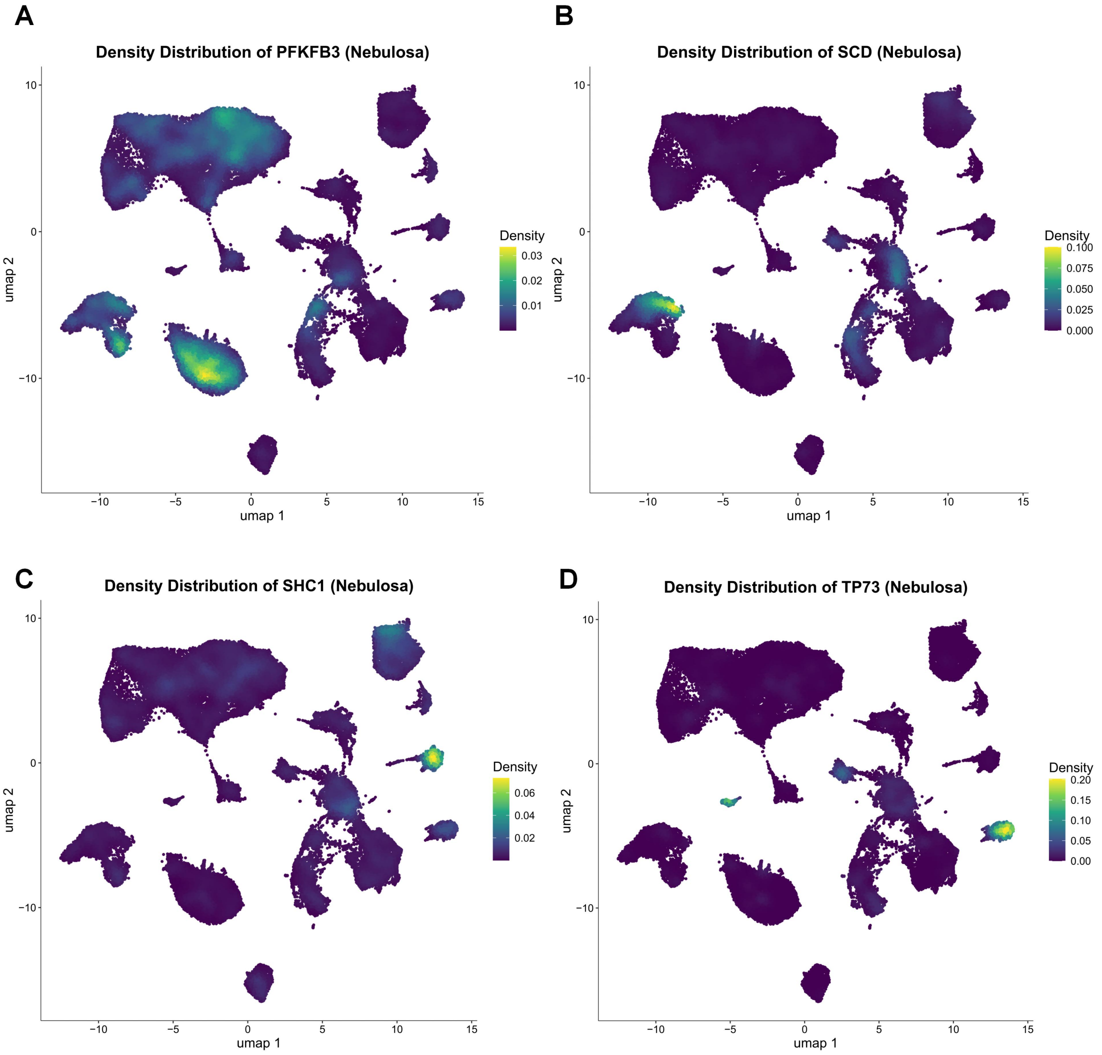

Supplementary Figure S5. Nebulosa-based expression density plots of candidate prognostic genes. (A) Nebulosa-based expression density plot revealing the enrichment of PFKFB3, showing predominant and specific expression within the neutrophil population compared to other immune or stromal compartments. (B) Nebulosa-based expression density plot of SCD revealing its specific expression within the myeloid cell compartment. (C) Nebulosa-based expression density plot of SHC1 illustrating predominant localization within the endothelial cell population. (D) Nebulosa-based expression density plot revealing the specific enrichment of TP73, showing predominant expression within the epithelial cell population compared to immune or stromal compartments.

Supplementary Table S1: Primer sequences for RT-qPCR

Gene symbol	Forward primer (5' → 3')	Reverse primer (5' → 3')
CD86	TACACGGTTACCCAGAACCT	CCGCGTCTTGTCAGTTTCCA
CD206	TGGTGAACGGAATGATTGTGTAG	GGTCCATCTTCCTTGTGTCAG
iNOS	TCACCTACTTCCTGGACATCAC	GAACTTCCACTTGCTGTACTCTG
Arg-1	GGAAGACACCAGAAGAAGTAACTC	GGTTAAGGTAGTCAATAGGCTTGT

Notes: Abbreviations: RT-qPCR, real-time quantitative polymerase chain reaction; iNOS, Inducible nitric oxide synthase; Arg-1, Arginase 1.

Original pictures of Figure 8B

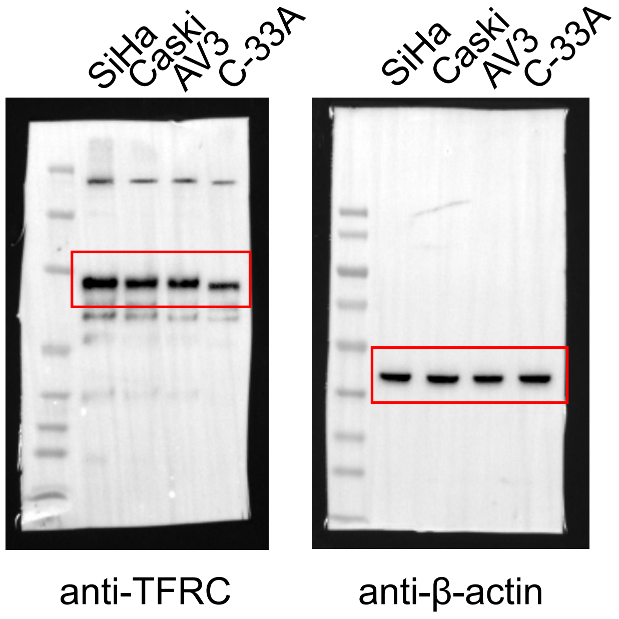

Original pictures of Figure 8C-E

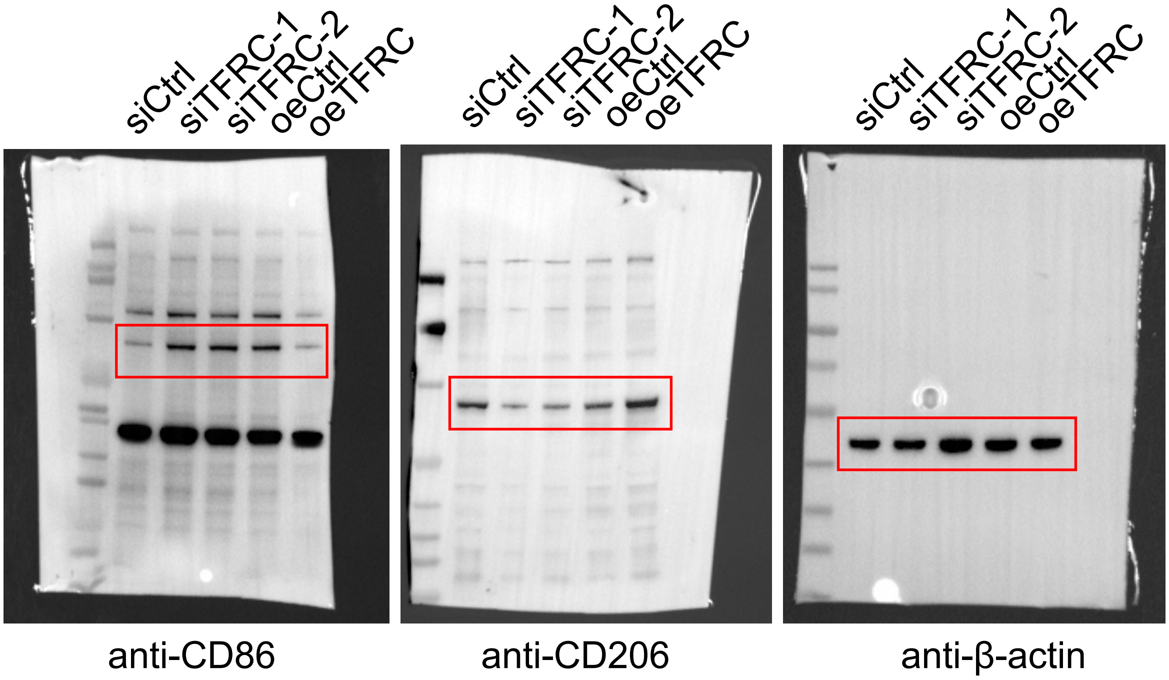

Supplement: Supplementary file 1 [file DataSheet1.pdf]
